# Eosinophilic Angiocentric Fibrosis: An Uncommon Entity in the Spectrum of IgG4-related Disease

**DOI:** 10.1007/s12105-025-01795-x

**Published:** 2025-05-26

**Authors:** Sarah E. Gradecki, Edward B. Stelow

**Affiliations:** Department of Pathology, University of Virginia, 1215 Lee Street, PO Box 800214, Charlottesville, VA 22908 USA

**Keywords:** Eosinophilic angiocentric fibrosis, Eosinophilia, IgG4-related disease, Head and neck, Upper aerodigestive tract

## Abstract

**Background:**

Eosinophilic angiocentric fibrosis (EAF) is a rare, tumefactive inflammatory process of the sinonasal and upper respiratory tract and orbit. Numerous histologic mimickers of EAF occur in these locations.

**Methods:**

A comprehensive literature review focused on EAF and its histologic mimickers was performed.

**Results:**

This manuscript serves as an overview of the histopathological characteristics of EAF and its histologic mimickers, including granulomatosis with polyangiitis (GPA), eosinophilic granulomatosis with polyangiitis (EGPA), sinonasal inflammatory polyps, epithelioid hemangioma, invasive fungal rhinosinusitis, Langerhans cell histiocytosis, rhinoscleroma, inflammatory myofibroblastic tumor, and fibromatosis.

**Conclusion:**

A correct diagnosis of EAF can be reached by identifying the classic histopathologic features of prominent eosinophilia, lymphoplasmacytic inflammation, and concentric perivascular fibrosis. Additionally, as EAF sometimes lies on the spectrum of IgG4-related disease, immunohistochemistry and serologic studies can be used to aid in diagnosis.

## Background

Eosinophilic angiocentric fibrosis (EAF) is a rare, indolent, tumefactive condition that most commonly involves the sinonasal and upper respiratory tract and orbit [1–3]. First described in 1983 as an intranasal variant of granuloma faciale due to its histologic similarity to the cutaneous inflammatory process of the same name, this entity was given its current name in 1985 in the report of 3 cases by Roberts and McCann [4, 5]. Since then, approximately 84 cases of EAF have been described in the literature. A chronic and infiltrative process, EAF is benign but difficult to manage clinically, with recurrences and multiple surgical interventions likely throughout the course of the disease [3, 6, 7]. While the etiology of this condition is still under investigation, at least a subset of cases appear to be associated with IgG4-related disease (IgG4-RD) [8].

## Clinical Features

EAF typically presents as a submucosal lesion in the sinonasal tract following a prolonged history of nasal swelling and obstructive symptoms [6, 7]. The disease shows a slight female predilection, and patients are typically adults ranging in age from 16 to 79 years [3, 8–10]. The clinical symptoms precede a tissue diagnosis by months to up to 20 years [3, 6, 11]. Most cases show involvement of the anterior nasal cavity, involving the nasal septum or lateral nasal wall [7]. More uncommon primary sites of involvement include the lower respiratory tract and orbit [12–17]. The majority of sinonasal lesions present on imaging studies as ill-defined soft-tissue thickening that may progress to show adjacent bone changes including remodeling, thinning, and sclerosis. True bone destruction is absent [7].

### Histopathologic Features

**Fig. 1:**
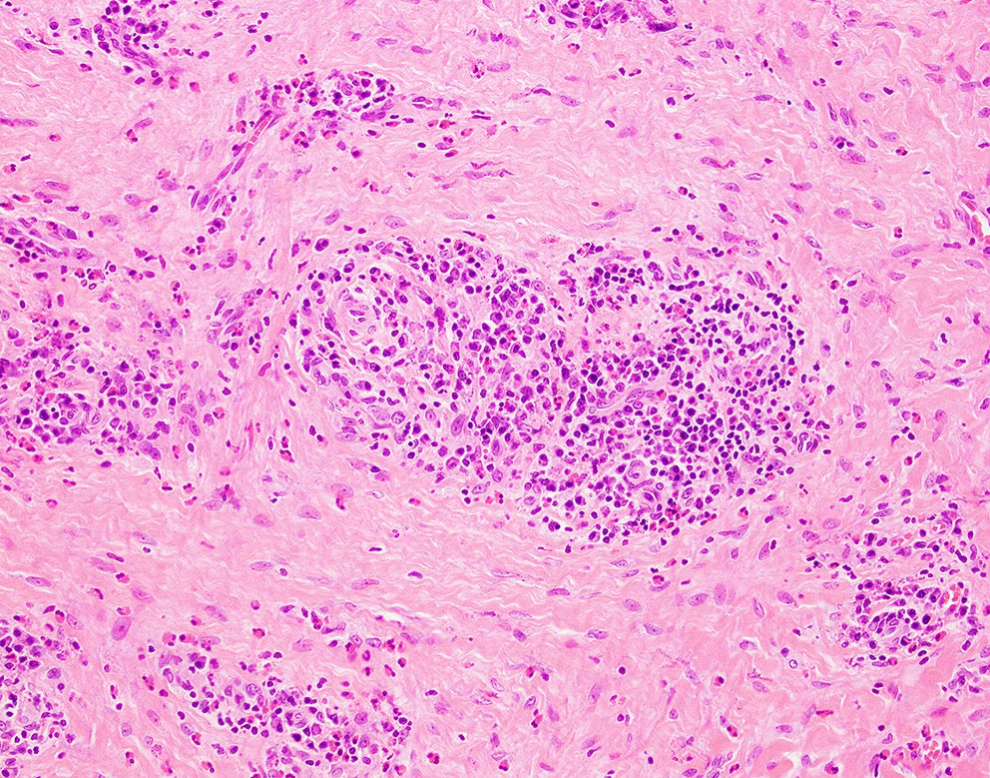
A 23-year-old woman who presented with a 9-month history of increasing nasal obstruction was found to have an anterior septal perforation with a scar-like mass obstructing the nares. Biopsy showed fibrotic stromal tissue with numerous eosinophils and plasma cells concentrated around small vessels

**Fig. 2:**
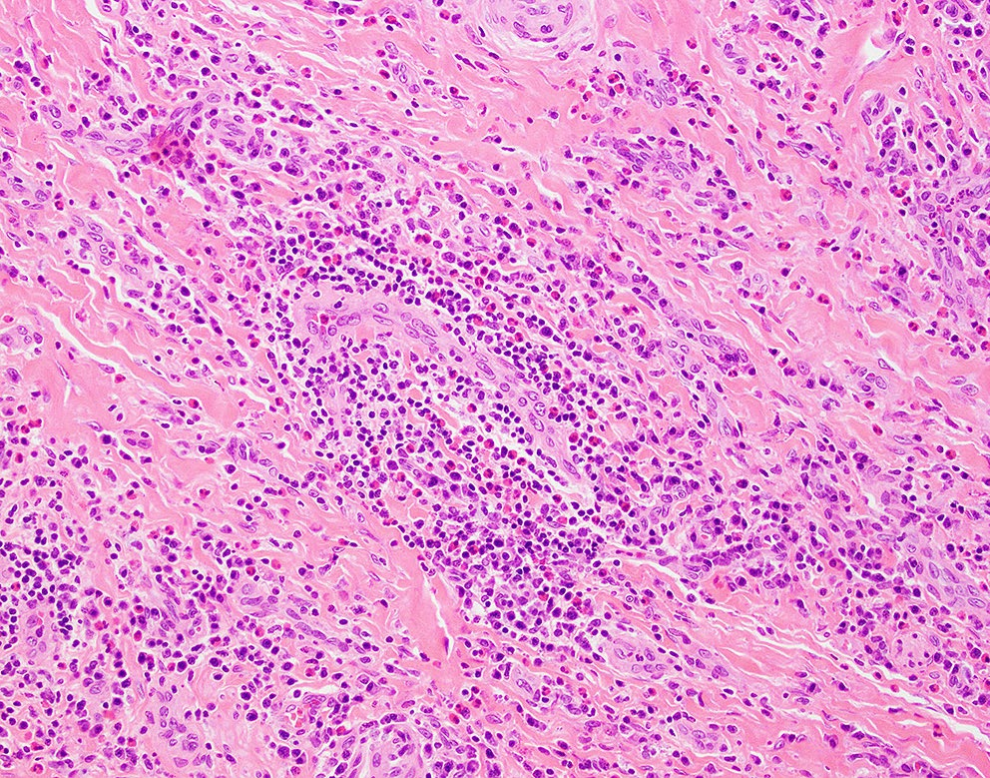
Biopsy from the same patient as in Fig. 1 showing similar findings in another sample of the mass

**Fig. 3:**
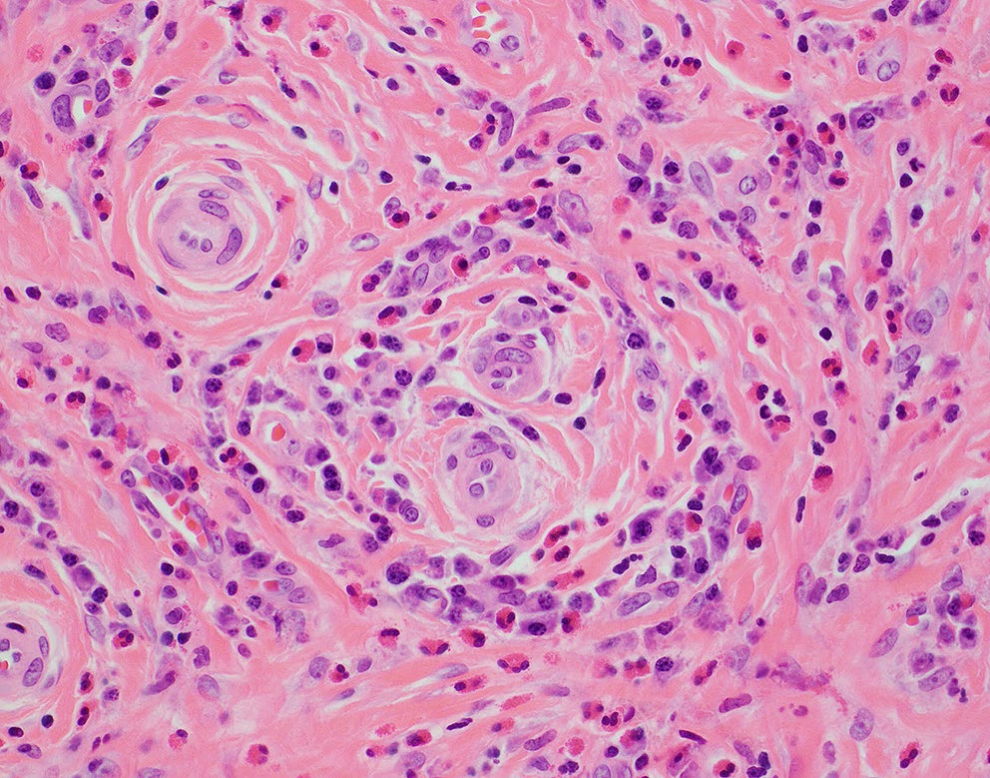
Biopsy from the same patient as in Fig. 1 with a high-power image showing the perivascular nature of the infiltrate without definitive vasculitis

Microscopically, EAF demonstrates concentric (“onion skin”) perivascular fibrosis with a mixed inflammatory infiltrate containing numerous eosinophils (see Figs. 1, 2 and 3) [3]. However, the histologic features vary in type and extent depending on the clinical age of the lesion. Early lesions show denser inflammation comprising eosinophilic and lymphoplasmacytic infiltrates that vary in intensity depending on the age of the lesion. While the literature does not reflect consensus on whether true eosinophilic vasculitis is present, it is accepted that there may be significant eosinophilic infiltration into small, submucosal vessels [6]. Lymphoid follicles may or may not be present. As the lesions progress, concentric lamellar fibrosis becomes a more prominent feature while the lymphoplasmacytic infiltrate typically wanes in density. Eosinophils remain a prominent inflammatory cell type even as lesions show increasing fibrosis (see Table 1). True granulomatous inflammation, infectious organisms, and necrosis are not features of EAF.^6^ While obliterative phlebitis at many anatomic sites has been reported in patients with IgG4-RD, this finding has not been identified in EAF.^6^.

**Table 1:** Histologic features of eosinophilic angiocentric fibrosis

Early	Late
Dense inflammatory lymphoplasmacytic inflammatory infiltrate with eosinophils	Sparse inflammation with prominent eosinophils
Eosinophils within submucosal capillary walls	Perivascular concentric lamellar fibrosis

**Table 2:** Histologic mimickers of eosinophilic angiocentric fibrosis

Entity	Distinguishing Features
GPA/EGPA	Granulomatous inflammation Necrosis
Sinonasal inflammatory polyp	Subepithelial edema Hyalinization of basement membrane
Angiolymphoid hyperplasia with eosinophilia	Capillary proliferation with epithelioid endothelial cells Lacks fibrosis
Invasive fungal rhinosinusitis	Fungal organisms Necrosis Dense neutrophilic infiltrate
Langerhans cell histiocytosis	Histiocytes with reniform nuclei Immunohistochemical evidence of Langerhans cells (S100^+^, CD1a^+^, Langerin^+^)
Rhinoscleroma	Mikulicz cells Gram-negative bacilli Absence of significant eosinophilia
Inflammatory myofibroblastic tumor	Myxoid stroma Ganglion-like myofibroblasts *ALK* gene rearrangement

Abbreviations: GPA, granulomatosis with polyangiitis; EGPA, eosinophilic granulomatosis with polyangiitis

## Etiology and Relationship To IgG4-Related Disease

EAF is of unclear etiology and has historically been attributed to trauma, prior surgery, and hypersensitivity/atopy [6]. EAF was first proposed to be a manifestation of IgG4-RD in 2011, but since that time multiple cases have been reported that do not show diagnostic features of IgG4-RD [8, 11]. However, at least a minority of cases meet the 2019 American College of Rheumatology/European League Against Rheumatism (ACR/EULAR) classification criteria for IgG4-RD, suggesting that patients who receive a diagnosis of EAF should be histopathologically and clinically evaluated for IgG4-RD [6, 8].

**Fig. 4:**
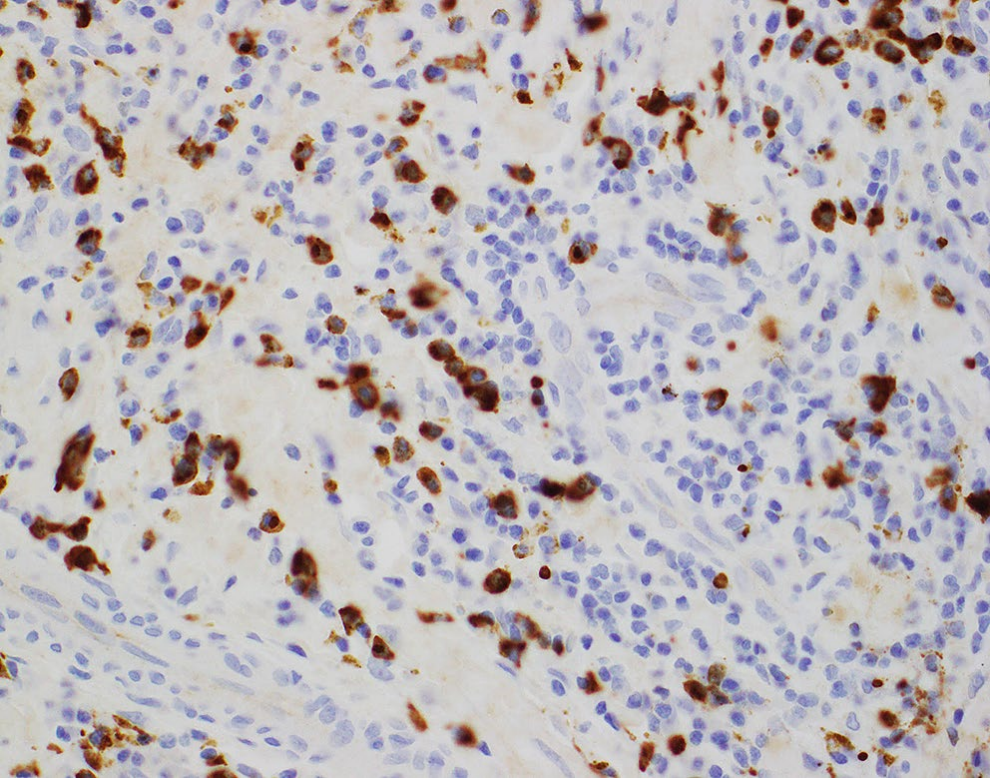
Biopsy from the same patient as in Fig. 1 showing IgG4 immunohistochemical stain highlighting numerous plasma cells

Given the association of EAF with IgG4-RD, microscopic analysis of biopsies from patients with suspected EAF should include immunohistochemical staining for IgG and IgG4. Infiltrates of IgG4^+^ plasma cells numbering over 50 per high-power field and/or an IgG4^+^/IgG^+^ plasma cell ratio greater than 40% are highly specific for the diagnosis of IgG4-RD (See Fig. 4) [18]. These immunohistochemical findings seen in association with elevated serum IgG4 levels, a tumefactive lesion with subacute onset, involvement of multiple organs, and rapid clinical response to immunosuppressive therapy strongly support a diagnosis of IgG4-RD [19].

## Differential Diagnosis

Given the variability in the histologic appearance of EAF according to the age of the lesion, diagnosis can be challenging, and several other entities occurring in the upper aerodigestive tract should be considered in the histologic differential diagnosis; specifically, granulomatosis with polyangiitis (GPA), eosinophilic granulomatosis with polyangiitis (EGPA), sinonasal inflammatory polyps, epithelioid hemangioma, invasive fungal rhinosinusitis, Langerhans cell histiocytosis, rhinoscleroma, inflammatory myofibroblastic tumor, and fibromatosis (see Table 2).

GPA and EGPA are systemic vasculitides characterized by small-vessel vasculitis, necrosis, and granulomatous inflammation. EAF can be distinguished from GPA and EGPA by the absence of necrosis and granulomatous inflammation. Clinical serologic studies can also be used in evaluation of these entities, as most patients with GPA have PR3-ANCA, and a significant minority of patients with EGPA have MPO-ANCA [20, 21]. It is important to note that an elevated serum IgG4 can be seen in up to 75% of patients with EGPA [22]; however, the peripheral eosinophilia typical of EGPA has not been reported in patients with EAF [21].

Sinonasal inflammatory polyps share some histologic features with EAF, specifically, their eosinophil-rich, mixed inflammatory infiltrate. However, characteristic findings of sinonasal inflammatory polyps include subepithelial edema and hyalinization of the basement membrane, neither of which are seen in EAF [23]. Furthermore, inflammatory polyps typically occur as multiple polypoid lesions throughout the sinonasal tract in patients with allergic disease and do not present as enlarging mass lesions.

Angiolymphoid hyperplasia with eosinophilia (ALHE), also known as epithelioid hemangioma, is characterized by a vaguely lobular capillary proliferation composed of epithelioid endothelial cells with abundant eosinophilic cytoplasm. There is a background of dense lymphocytic inflammation and admixed eosinophils. The epithelioid morphology of the endothelial cells and lack of notable fibrosis help to differentiate ALHE from EAF [24].

Invasive fungal rhinosinutitis (IFRS) typically has a more rapid clinical onset than EAF and invariably shows necrosis and tissue invasive fungal forms on histologic evaluation. As compared with EAF, IFRS exhibits a more scant inflammatory response, as patients are typically immunocompromised, and fibrosis is absent [25].

Langerhans cell histiocytosis (LCH) characteristically shows a dense mononuclear inflammatory cell infiltrate with admixed eosinophils, which could potentially mimic the mixed chronic and eosinophilic inflammation of EAF. However, LCH typically lacks a significant lymphoplasmacytic component, and instead is composed of histocytes with abundant eosinophilic cytoplasm, reniform nuclei, and a typical immunophenotype (S100^+^, CD1a^+^, and Langerin/CD207^+^). Fibrosis is typically absent [26].

Rhinoscleroma may show overlapping histologic features with EAF, particularly in more chronic stages of infection. While early stages of rhinoscleroma demonstrate a dense inflammatory infiltrate composed of abundant foamy macrophages (“Mikulicz cells”) containing gram-negative bacilli with an associated plasma cell population, later stages may show more overlap with EAF due to significant fibrosis.^27^ At this stage, both rhinoscleroma and EAF may be pauci-inflammatory, but typically EAF retains an eosinophilic infiltrate [6].

Inflammatory myofibroblastic tumor (IMT), which occurs very rarely in the sinonasal tract, is characterized by a proliferation of spindled myofibroblasts, some of which have a ganglion-cell like appearance with eccentric nuclei, vesicular chromatin, and small nucleoli. The background stroma is often myxoid, and there is a brisk associated inflammatory infiltrate comprised of lymphocytes, plasma cells, neutrophils and eosinophils. While both IMT and EAF can show an inflammatory infiltrate, the atypical spindle cell/ganglion-like cell population of IMT is absent in EAF. Additionally, the majority of IMT cases harbor anaplastic lymphoma kinase (*ALK*) gene rearrangements [9].

While other lesions with a prominent fibrous stroma including angiofibroma and fibromatosis may arise in the differential diagnosis for EAF in the later stages, neither of these entities has a notable inflammatory infiltrate.^9^.

## Treatment and Prognosis

Surgical excision is the mainstay of treatment for EAF, with either complete excision or debulking [6]. Systemic and intralesional corticosteroid therapy are also used; however, patients who undergo complete surgical excision have the best outcomes regardless of medical therapy. In a number of cases arising in the setting of IgG4-RD, rituximab has shown efficacy.^11^ Due to the anatomic location and consequent difficulty in achieving complete resection of these lesions, they have a high risk of recurrence, and patients require long-term follow-up. However, malignant transformation has not been reported in EAF [6].

## Conclusion

EAF is a rare, progressive but indolent condition involving the sinonasal and upper respiratory tract and orbit that requires histologic evaluation for diagnosis given overlapping radiologic features with other benign and malignant processes. As this diagnosis is sometimes associated with IgG4-RD, additional clinical workup is recommended for definitive diagnosis and to assess for involvement of other organs. Accurate diagnosis of EAF is crucial, as the primary management of EAF is surgical excision, while some histologically similar entities require systemic therapies.

## Data Availability

No datasets were generated or analysed during the current study.

## Code Availability

No custom code was used in preparation of this article.
